# Complete Genome Sequences of Multidrug-Resistant *Campylobacter jejuni* Strain 14980A (Turkey Feces) and *Campylobacter coli* Strain 14983A (Housefly from a Turkey Farm), Harboring a Novel Gentamicin Resistance Mobile Element

**DOI:** 10.1128/genomeA.01175-16

**Published:** 2016-10-20

**Authors:** William G. Miller, Steven Huynh, Craig T. Parker, Jeffrey A. Niedermeyer, Sophia Kathariou

**Affiliations:** a Produce Safety and Microbiology Research Unit, Agricultural Research Service, U.S. Department of Agriculture, Albany, California, USA; b Department of Food, Bioprocessing and Nutrition Sciences, North Carolina State University, Raleigh, North Carolina, USA

## Abstract

Multidrug resistance (MDR) in foodborne pathogens is a major food safety and public health issue. Here we describe whole-genome sequences of two MDR strains of *Campylobacter jejuni* and *Campylobacter coli* from turkey feces and a housefly from a turkey farm*.* Both strains harbor a novel chromosomal gentamicin resistance mobile element.

## GENOME ANNOUNCEMENT

*Campylobacter jejuni* and *C. coli* are leading bacterial agents for human foodborne disease, with poultry serving as a major vehicle (1). They frequently exhibit resistance to various antimicrobials, especially tetracycline (2). Resistance to the aminoglycoside gentamicin was rare, but has recently increased markedly (http://www.cdc.gov/narms/pdf/2011-annual-report-narms-508c.pdf, http://www.fda.gov/downloads/AnimalVeterinary/SafetyHealth/AntimicrobialResistance/NationalAntimicrobialResistanceMonitoringSystem/UCM453387.pdf). Here we report the whole-genome sequence of *C. jejuni* strain 14980A, isolated in July 2014 from turkey feces in a commercial turkey house (conventional production) in North Carolina, and *C. coli* strain 14983A, isolated on the same date from a housefly (*Musca domestica*) in the same turkey house. The fly isolate *C. coli* 14983A was resistant to all tested antibiotics (tetracycline, streptomycin, erythromycin, kanamycin, ciprofloxacin, nalidixic acid, gentamicin) while *C. jejuni* strain 14980A was resistant to all but erythromycin.

The Roche GS-FLX, Illumina MiSeq and PacBio RSII platforms were used to complete both genomes. Roche 454 shotgun and paired-end reads were assembled into single chromosomal scaffolds using the Roche Newbler assembler (v2.6); however, neither scaffold could be closed due to chromosomal repeats. Therefore, PacBio sequencing was used to generate single contigs for both genomes, and also generated single contigs for both megaplasmids. A final base call validation was performed using Illumina MiSeq reads. Illumina reads were also used to characterize hypervariable GC tracts, as described (3). The final coverage for both strains was >200×.

The two strains have circular genomes of 1,797 kb (14983A) and 1,709 kb (14980A). *C. coli* strain 14983A harbors three plasmids (180.5, 4.4, and 3.1 kb) and has sequence type (ST) 1067, while *C. jejuni* strain 14980A contains a single plasmid (50.7 kb) and has ST-1839. Protein-, rRNA-, and tRNA-encoding genes were identified as described (3), using a BLASTp identity of 50% to define a positive protein match. Nine predicted functional/degenerate IS elements were identified in *C. coli* strain 14983A and two in *C. jejuni* strain 14980A.

Genes or point mutations associated with the antibiotic resistance profiles were identified. Both megaplasmids harbor *tet(O)* associated with tetracycline resistance (4), along with either *aphA-3* (*C. jejuni* strain 14980A) or *aphA-7* (*C. coli* strain 14983A) that confers kanamycin resistance (5–7). Ciprofloxacin and nalidixic acid resistance in both strains was due to a Thr 86 → Ile substitution within GyrA (8, 9). Similarly, streptomycin resistance was associated with a substitution within RpsL: Lys 43 → Arg in *C. coli* strain 14983A and Lys 88 → Arg in *C. jejuni* strain 14980A (10, 11). Erythromycin resistance in *C. coli* strain 14983A was conferred by an A → G transition in all three copies of the 23S rRNA gene (equivalent to A2059 → G in *Escherichia coli* [12, 13]). An unique characteristic of both strains was a novel IS*1595*-family chromosomal mobile element, inserted at a different site in each strain (linked to *fabG* in *C. jejuni* strain 14980A and *rplM* in *C. coli* strain 14983A), harboring the gentamicin resistance determinant *aph(2′′)-If* (14). Although the element in 14983A also harbored an IS*605*-family element, the two elements were essentially identical (2-bp divergence).

### Accession number(s).

The complete genome/plasmid sequences of *C. coli* strain 14983A and *C. jejuni* strain 14980A have been deposited in GenBank under the accession numbers CP017025 to CP017028 and CP017029 to CP017030, respectively.
